# Anti-proliferative activity, molecular genetics, docking analysis, and computational calculations of uracil cellulosic aldehyde derivatives

**DOI:** 10.1038/s41598-023-41528-0

**Published:** 2023-09-04

**Authors:** Asmaa M. Fahim, Sawsan Dacrory, Ghada H. Elsayed

**Affiliations:** 1 Green Chemistry Department, National Research Centre (NRC), P.O. Box 12622, DokkiCairo, Egypt; 2 Cellulose and Paper Department, National Research Centre, P.O. Box 12622, Giza, Egypt; 3 Department of Hormones, National Research Centre (NRC), P.O. Box 12622, Dokki, Giza, Egypt; 4 Stem Cells Lab, Center of Excellence for Advanced Sciences, National Research Centre (NRC), P.O. Box 1262, Dokki, Giza, Egypt

**Keywords:** Cancer, Chemical biology, Chemistry, Physics

## Abstract

In this study, the oxidation of microcrystalline cellulose using NaIO_4_ to yield the corresponding cellulose aldehyde utilized microwave irradiation as a green tool, the obtained cellulosic aldehyde was confirmed through spectral analysis and it has an active site to react with the synthesized uracil acetamide to afford the corresponding arylidene cellulosic MDAU(4), the latter compound which can easily due to presence of active CH=group behind a cyano group react with nitrogen nucleophile’s and cyclized with hydrazine hydrate to give pyrazole cellulosic MDPA(5). The spectral analysis of the obtained cellulosic derivatives was confirmed with FT-IR, NMR, and SEM. Additionally, a neutral red uptake analysis has been used to investigate the cytotoxic activity of the cellulosic compounds MDAC(2), MDAU(4), and MDAP(5) against the cancer cells A549 and Caco2. After 48 h, Compound MDAU(4) had a stronger inhibitory effect on the growth of A549 and Caco2, compared to control cells. Then, using QRT-PCR, the expression levels of the genes β-Catenin, c-Myc, Cyclin D1, and MMP7 in A549 cells were examined. By reducing the expression levels of the Wnt signaling cascade genes (β-Catenin, c-Myc, Cyclin D1, and MMP7) when administered to A549 cells, compound MDAU(4) was shown in this investigation to be a viable candidate compared to lung cancer. Additionally, docking simulation was used to explore the uracil cellulosic heterocycles attached to different proteins, and computational investigations of these compounds looked at how well their physical characteristics matched the outcomes of their experiments.

## Introduction

The waste minimization of agriculture waste takes the attention of most scientists due to the presence of cellulosic bone which has chains comprised β(1 → 4) linked to D-glucose^1–5^ and have different hydrogen bonding interactions, that are active in biological estimation for instance bio adhesive drug delivery systems^3,6–8^. Furthermore, Lung cancer is one of the maximum popular cancers internationally, and it is disturbing in males and females^9,10^. Wnt/β-catenin signaling pathway is an important molecular cascade in modified embryonic development and adult homeostasis^11–14^. However, it has been implicated in multiple human malignancies, including colorectal cancer, breast cancer, prostate cancer, and non-small cell lung cancer, due to dysregulation^15–18^. Beta-catenin is one of a big and complicated array of proteins which it's involved in the control of the Wnt/β-catenin signaling pathway^18,19^. Also, the dissociation of beta-catenin from this destruction complex outcomes in the aggregation of beta-catenin in the cytoplasm and nucleus, which activates the target genes of the Wnt pathway, such as cyclin D1, MMP7, and c-Myc in human cancers^20,21^. In this elucidation, we synthesized the novel pyrazole cellulosic compounds from the reaction of condensation with cellulose aldehyde with active CN-uracil acetamide to give the corresponding cellulose arylidene **MDAU(4).** This compound can then be easily cyclized in the presence of nitrogen nucleophiles like hydrazine hydrate to give the pyrazole cellulosic derivative **MDAP(5),** which was confirmed through spectral analysis. Additionally, we examined these cellulosic derivatives with cytotoxic effects on lung cancer cells (A549) and colon cancer cells (Caco2), and we then examined the levels of β-Catenin, c-Myc, Cyclin D1, and MMP7 gene expression in A549 cells. The heterocyclic cellulosic compounds were also docked with the **PDBID:**2ito, 1nun, 5jsn, and 5vzu, respectively. Additionally, the cellulosic compounds were improved utilized the DFT/B3LYP/6-311(G) basis set, which demonstrated the stability of the compounds as a result of the interaction of the NH and OH of cellulose, uracil, and pyrazole in electrostatic hydrogen bonds^14,22–24^.

## Experimental section

### Apparatus

The Shimadzu FT-IR 8101 PC infrared spectrophotometer recorded the IR spectra. The ^1^H NMR and ^13^C NMR spectra were determined in DMSO-d_6_ at 300 MHz on a Varian Mercury VX 300 NMR spectrometer (^1^H at 300 MHz, ^13^C at 75 MHz) exhausting trimethyl silane as an internal typical. Scanning electron microscopes (SEM) were investigated utilizing JEOL JXA-840A electron probe Microanalyzer Company and were air-dried before imaging, and images were obtained using an accelerating voltage of 10–15 kV. In closed vessels under pressure, microwave-irradiated Pyrex tubes with caps were used to conduct the reactions.

### Reagent

Microcrystalline cellulose (20 μm) was obtained from the Rasyan research laboratory, and NaIO4 was ordered from Fluka. EtOH, NH_2_NH_2_, triethyl amine, purchased from Aldrich Chemical Company. 2-cyano-*N*-(2,4-dioxo-1,2,3,4-tetrahydropyrimidin-5-yl)acetamide(3) was synthesized according previous procedure^25^.

### Synthesis of cellulose aldehyde MDAC(2)

Using a microwave device, cellulose was oxidized to various degrees of oxidation with NaOI4 (20 ml). At 3, 1, and 1.5 min, the precursor was transferred to the microwave to finish the oxidation process. The oxidized product was filtered, EtOH washed, and allowed to dry for a whole night at room temperature. Using 1.5 g of cellulose and 20 ml of distilled water, aldehyde concentration was determined^26^, through the basic response of Schiff. NH_2_OH.HCl is used when aldehyde groups transform into oximes. Dialdehyde cellulose, 0.3 g, was dispersed in 20 ml of pH-5 NaOH solution-containing water, then, NH_2_OH.HCl (0.72 mol/l) was added at pH 5 for 4 hours, the mixture was stimulated at 40 °C. To titrate the emitted HCl, a 1.0 M aqueous NaOH solution was used. The volume of alkali solution consumed during the titration was recorded (in liters), and the amount of NaOH consumed when the pH value of the solution reached 5.0 was validated. The volume of 1.0 M sodium hydroxide solution consumed was measured as Vc (in liters), using the same concentration of cellulose solution at pH 5.0 as a blank. The following equation is used to designate the aldehyde content (% w/w)^27,28^.

$$\begin{aligned} & {-}CHO + {\text{NH}}_{{2}} {\text{OH}} \to {-}{\text{CHNOH}} + {\text{HCL}} + {\text{H}}_{2} {\text{O}} \\ & {\text{HCL}} + {\text{NaOH}} \to {\text{NaCL}} + {\text{H}}_{{2}} {\text{O}} \\ \end{aligned}$$

$$Aldehyde \, content\left( \% \right)t = \frac{{{\text{C}}({\text{NaOH}}) \cdot Va - VC}}{{8\;{\text{mM}}}}$$

where C_NaOH_ = 1.0 M, m is the dry weight of DAC (0.3 g) used in the experiment, and M molecular weight of the repeating unit of cellulose (162).

### Reaction of MDAC(2) with synthesized 2-cyano-*N*-(2,4-dioxo-1,2,3,4-tetrahydropyrimidin-5-yl)acetamide(3)

Stirring solution of MDAC(**2**) (1 g, 10 mmol) was mixed with 2-cyano-*N*-(2,4-dioxo-1,2,3,4-tetrahydropyrimidin-5-yl)acetamide(**3**)(1 g, 10 mmol) in EtOH and 2 drops of triethylamine as basic catalyst were refluxed for 5 h at 120 °C and monitored through TLC with (ethyl acetate/petroleum ether) eluent(1:1) then the product filtered off and recrystallized from EtOH/H_2_O to afford the corresponding ***(Z)-2-cyano-N-(2,4-dioxo-1,2,3,4-tetrahydropyrimidin-5-yl)-5,7-dihydroxy-6-(1-hydroxy-2-oxoethoxy)hept-2-enamide (4)***: colorless powder, m.p =  > 300 °C, FT-IR (KBr): ν 3410(OH), 3352(NH), 3200(NH), 3174(NH), 2990 (CH_2_), 1650(C=O),1550(C=C); ^1^H-NMR (DMSO-d6): δ 3.54(m, CH_2_, glygose), 4.02(m, OH), 6.6 (d, H, CH), 7.02(d, H, CH),7.9 (H, s, CH,), 7.5 (m, H, CH), 8.96 (s, NH uracil, D_2_O exchangeable),10.32(s, CH=) 11.62(NH, D2O exchangeable), ^13^CNMR(DMSO-d6), δ 28(CH_2_), 66(CH_2_), 77(CH_2_), 88(CH_2_), 100 (CH=), 110(CH), 115(CN), 134(CH),150(CH=),160(C=O), 169(CH=).

### Synthesis of MDUP(5)

Reactivity of MDAU(**4**) (0.368 g, 1 mmol) with hydrazine hydrate (1 mmol) was heated for 6 h, then left to cool and monitored through TLC with (ethyl acetate/petroleum ether) eluent (1:!) then the product was formed and filtered off, washed with ethanol, and dried. Recrystallization from DMF/H_2_O to give 3***-amino-5-(1,3-dihydroxy-2-(1-hydroxy-2-oxoethoxy)propyl)-N-(2,4-dioxo-1,2,3,4-tetrahydropyrimidin-5-yl)-1H-pyrazole-4-carboxamide(5)*:** yellow powder, m.p =  > 300 °C, FT-IR (KBr): ν 3455(OH), 3400(OH), 3364(NH), 3305(NH), 3214(NH), 3204(NH), 3175(NH_2_), 2990 (CH_2_), 1715(C=O), 1655(C=O), 1599(C=O), 1510(C=C), ^1^H-NMR (DMSO-d6): δ 3.65(m, CH_2_, glycose), 4.11(s, OH, D2O exchangeable), 5.21 (s, OH, D_2_O exchangeable), 6.35 (d, NH_2_, D2O exchangeable), 7.5 (d, H, CH), 9.52 (1H, s, NH exchangeable),10.02(s, OH, D2O exchangeable),11.20 (s, NH D2O exchangeable), 11.52(s, NH, exchangeable), 12.01(s, NH, exchangeable), ^13^CNMR(DMSO-d6), δ 55(CH_2_), 84(CH2),100(CH_2_), 112 (CH),135(CH), 141(CH),155(C=O),160(C=O), 164(C=O), 177(C=O).

### Cytotoxicity screening

**A549** and **Caco2** cell lines were acquired from American Type Culture Collection and conserved in suitable conditions. **A549** cells were refined in DMEM (Dulbecco’s modified Eagle’s Medium) and **Caco2** cells were cultured in RPMI Medium containing 10% fetal bovine serum (FBS), 100 U/ml penicillin, and 100 μg/ml streptomycin sulfate at 37 °C in a humidified 5% CO_2_. The cells were digested with 0.025% trypsin-EDTA for passaging. The cells in the logarithmic growth phase were used in the experiment. The cytotoxicity of uracil pyrazole cellulosic compounds was examined via a neutral red uptake method. Several concentrations (6.25, 12.5, 25, 50, 100, and 200 µM) of heterocycles were added to continue the culture for 48 hours at a cell density of 10^4^ cells/well of 96 well plate and neutral red uptake assay was done as reported by Repetto et al.^29^. The relative between the used log concentrations and the neutral red intensity value accustomed estimate half maximal inhibitory concentration (IC_50_) of heterocycles. The medium was added instead of the cellulosic heterocycles for the untreated cells (negative control). A cytotoxic natural agent (doxorubicin, Mr = 543.5) was used as positive control giving 100% inhibition. Cellulose products were dissolved in DMSO and its final concentration was not increased than 0.2% in the cells. All analyses were achieved at least three times.

### Gene expression analysis

#### Quantitative real-time PCR

RNA was isolated from **A549** cells (3 × 10^4^ cells/well) and treated for 48 h using RNA easy mini Kit (Qiagen, USA) then the concentration and purity of total extracted RNA were determined using NanoDrop one micro-volume UV spectrophotometer (Thermos Fisher Scientific, USA). RNA of each treatment was converted to first-strand cDNA according to manufacturer instructions using Revert Aid First Strand cDNA Synthesis Kit (Thermo Scientific, USA). Specific primer sequences are listed in Table 1. Expression levels of *β-Catenin, c-Myc, Cyclin D1, and MMP7* genes were normalized concerning *β-actin* transcript using Maxima SYBR Green qPCR Master Mix (2X) (Thermo Scientific, USA) and calculated by 2^−ΔΔCT^ method^59^. The reaction conditions were as follows: 95 °C for 10 min, 95 °C for 15 s, 60 °C for 30 s and 72 °C for 30 s with a total of 40 cycles of amplification. DNA Technology Detecting Thermocycler DT Lite 4S1 was used for gene expression quantitation.

**Table 1:** Primer sequences used in the RT-PCR investigation.

Gene	Primer forward (5'-3')	Primer reverse (5'-3')
β-actin	CCTTCCTGGGCATGGAGTCCT	GGAGCAATGATCTTGATCTTC
β-Catenin	TAGAAACAGCTCGTTGTACCGCTGGGACCT	GCACTGCCATTTTAGCTCCTTCTTGATGTAAT
c-Myc	AGAGAAGCTGGCCTCCTACC	CGTCGAGGAGAGCAGAGAAT
Cyclin D1	GCTGCGAAGTGGAAACCATC	CCTCCTTCTGCACACATTTGAA
MMP7	GTGGTCACCTACAGGATCGTA	CTGAAGTTTCTATTTCTTTCTTGA

### Statistical evaluation

Outcomes are expressed as Mean ± SEM. Statistical analyses of the data are achieved utilizing Sigma plot ver. 125. A student t-test was used to analyses of the data and find the examined compounds’ significant differences. Changes were considered significant when *P* < 0.05.

#### Molecular docking studies

Dockage of uracil cellulosic heterocycles using the MOE program^30^ to confirm the biological action and communication with them concluded different binding proteins such as Crystal structure of EGFR kinase domain G719S mutation in complex with Iressa (**PDBID**:2ito)^31^, Crystal Structure Analysis of the FGF10-FGFR2b Complex (**PDBID**:1nun)^32^, Bcl2-inhibitor complex (**PDB ID:** (5jsn)^33^ and Crystal structure of the Skp1-FBXO31-cyclin D1 complex (**PDB ID**: (5vzu)^34^; correspondingly. For separate proteins, 9 dissimilar docking simulations were operate utilized default limits^3,35^.

#### DFT optimization

Uses of the gas phase of DFT/B3LYP/6-311(G) level utilized Gaussian 09 program^36^ was used for the geometry optimization^37,38^. The majority benefit of DFT methods is that they afford a substantial growth in computational accurateness lacking increasing computation time. All chemicals were imagined via Gauss-View^39^. Physical limitations were evaluated using the E_HOMO_ and E_LUMO_ values, while other limitations were retrieved from files^40,41^.

## Results section

### Chemistry part

**Figure 1:**
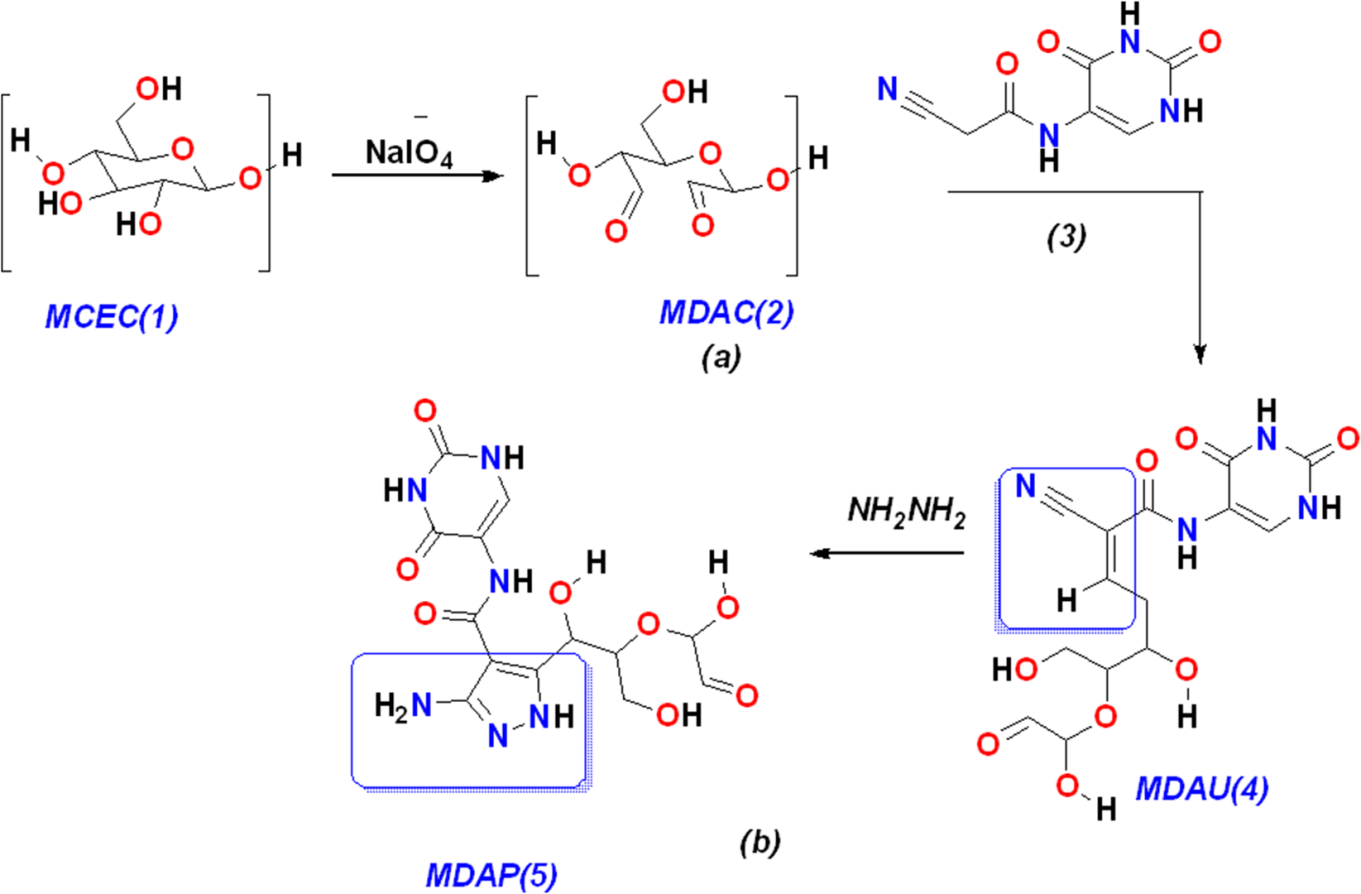
(**a**) Formation of cellulose aldehyde via oxidation (**b**) Reaction of MDAC with acetamide and formation of MDAP(**5**).

Oxidation of microcrystalline cellulosic ring MCEC(**1**) with sodium hypochlorite utilized Microwave irradiation MDAC(**2**) in excellent yield, the formation of cellulose aldehyde MDAC(**2**) was confirmed through previous studies^28,42^ and determination of cellulose aldehyde content, the reactivity of cellulose aldehyde MDAC(**2**) was so reactive which has methyl group which easily of attack with any active methylene group,  as displayed in Fig.1(a). Furthermore, the reaction of MDAC(**2**) with 2-cyano-*N*-(2,4-dioxo-1,2,3,4-tetrahydropyrimidin-5-yl)acetamide(**3**) in equal amounts to afford the corresponding (*Z*)-2-cyano-*N*-(2,4-dioxo-1,2,3,4-tetrahydropyrimidin-5-yl)-5,7-dihydroxy-6-(1-hydroxy-2-oxoethoxy)hept-2-enamide MDAU(**4**) and confirmed through FT-IR which showed 3410 cm^−1^ for hydroxyl group and more NH group at 3352-3174 cm^−1^ due to presence of uracil moiety and 2250 cm^−1^ for C≡N group, also the ^1^HNMR showed CH=olefin hydrogen of arylidene at δ = 10.32 ppm and more exchangeable proton at OH and NH of glycoside and uracil moieties at 4.02, 8.96, 11.62 ppm; respectively. Furthermore, the reactivity of arylidine MDAU(**4**) due to the presence of olefin hydrogen adjacent to the C≡N group which can be easy to cyclized in the presence of nitrogen nucleophiles such as NH_2_NH_2_, also the reactivity of MDAU(**4**) with hydrazine hydrate refluxing in EtOH to afford the corresponding 3-amino-5-(1,3-dihydroxy-2-(1-hydroxy-2-oxoethoxy)propyl)-*N*-(2,4-dioxo-1,2,3,4-tetrahydro pyrimidin-5-yl)-1*H*-pyrazole-4-carboxamide MDAP(**5**) and showed the stretching absorption band at 3175 cm^−1^ for an amino group of the pyrazole ring and more of NH bands at 3364–3204 cm^−1^ and the NMR showed exchangeable protons of NH_2_ group at 11.20 ppm and the NH protons were dishelded in 11.52, 12.01 ppm, and the ^13^CNMR showed the three carbonyl bonds from the range at 155–177 ppm; respectively as demonstrated in Fig. 1(b).

### IR characterization

**Figure 2:**
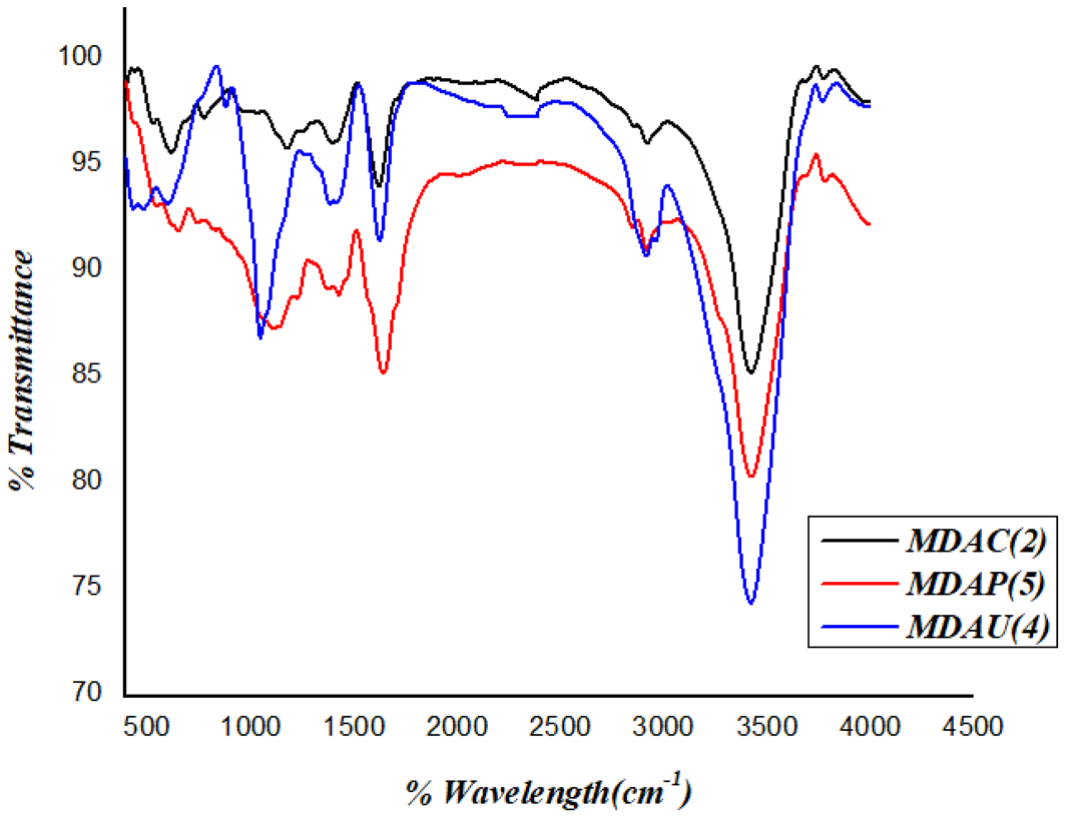
FT-IR of synthetized MDAC(2), MDAU(4) and MDAP(5); respectively.

FT-IR of the novel synthesized cellulosic heterocycles was cellulose aldehyde MDAC(2), arylidine MDAU(**4**), and Pyrazole cellulose MDAP(**5**) as displayed in Figure 2. The compound MDAC(**2**) showed a characteristic absorption band of C=O at 1650 cm^−1^ represents to two aldehyde group, and these bands can be so small or hidden in hydrated form but there is hemiacetal group which confirms the presence of aldehyde group. Furthermore, the presence of an adsorption band at 800–750cm^−1^ conforms to the hemiacetal bond which is a typical peak of aldehyde^26,28^. Moreover, the MDAU(**4**) showed a characteristic beak of the presence of three NH bands in the range 3352–3174 cm^−1^ due to uracil moiety, and the C≡N group showed bands at 2250 cm^−1^, carbonyl group at 1650 cm^−1^ and more CH stretching vibration at 2990 cm^−1^ for uracil and glycoside ring, also the MDAP(5) showed a wide range in OH vibration in 3455–3400 cm^−1^, and more bands for NH and NH_2_ which take the wide range in 3364–3175 cm^−1^ for amino pyrazole and uracil rings and cyano group is absent and there is more bending vibration for MDAU(4) and MDAP(5) for CH aliphatic of glucose and uracil and change the vibration of aldehyde cellulose.

### SEM analysis

**Figure 3:**
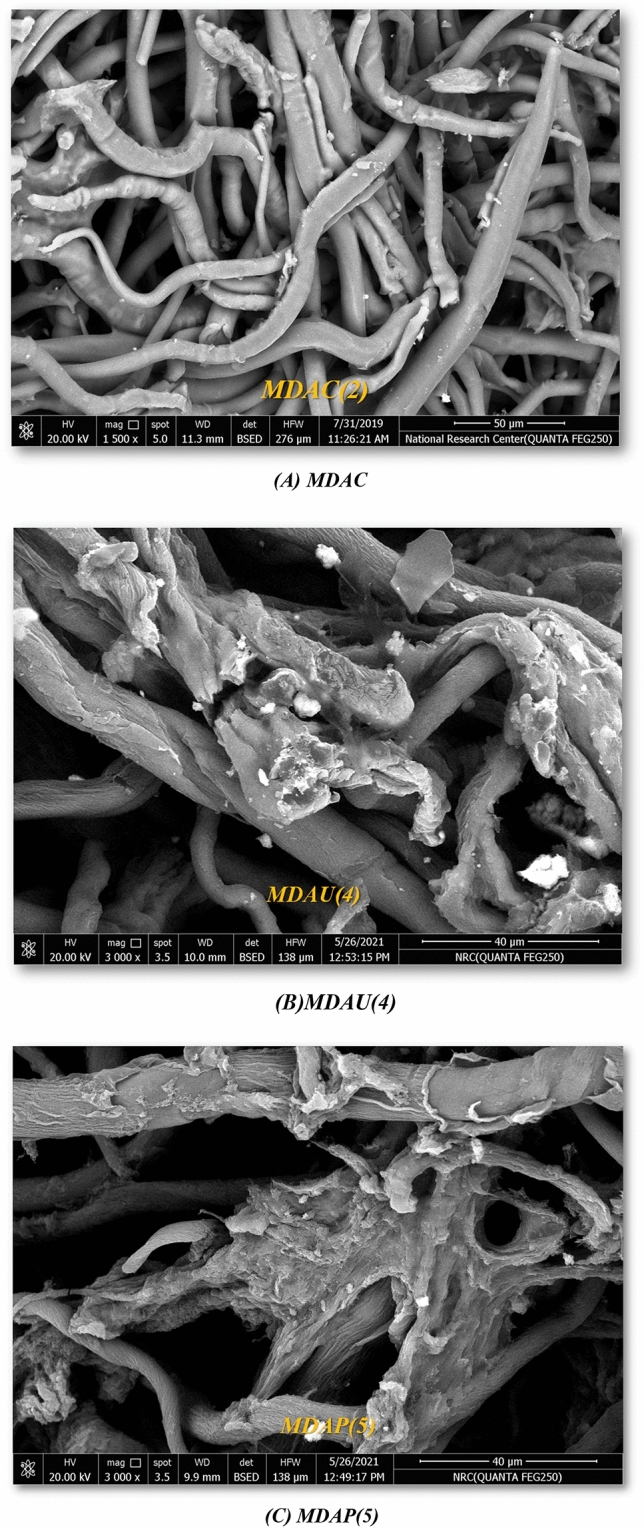
(**A**–**C**): SEM morphology of MDAC, MDAU and MDAP; respectively.

Additionally, SEM examination of cellulose aldehyde presented the tight bundles crossed to each other and it changed the morphology of cellulose due to the oxidation process as shown in Fig. 3A Furthermore, the reaction of MDAC(2) with uracil acetamide showed cracks on the surface of cellulose due to presence of more hydrogen bonds of NH and OH groups as showed in Fig. 3B and these cracks which bonded to each other’s again when reacted NH_2_NH_2_ to gave the pyrazole displayed its surface as the bundles collect to its self MDAP(5) as seen in Fig. 3C.

## Biological activities

### Anti-cancer activity

**Figure 4:**
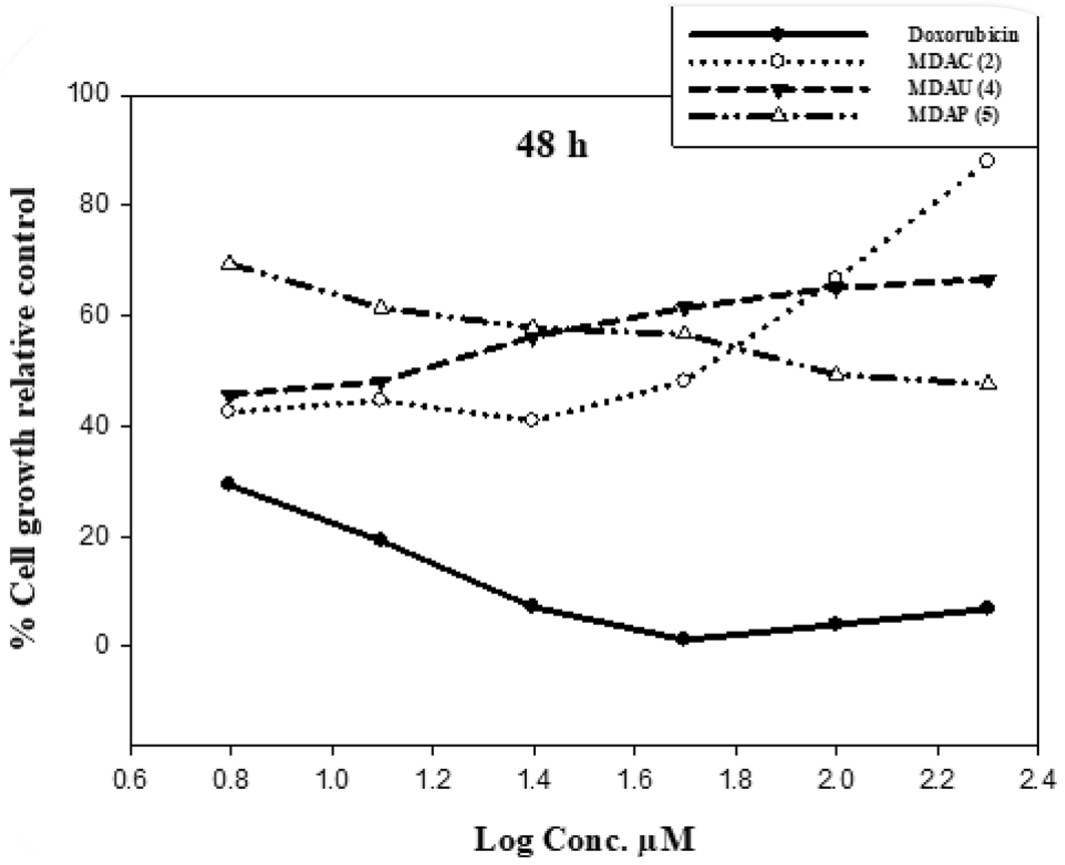
Outcome of cellulosic compounds on **A549** cells at 48 h.

**Figure 5:**
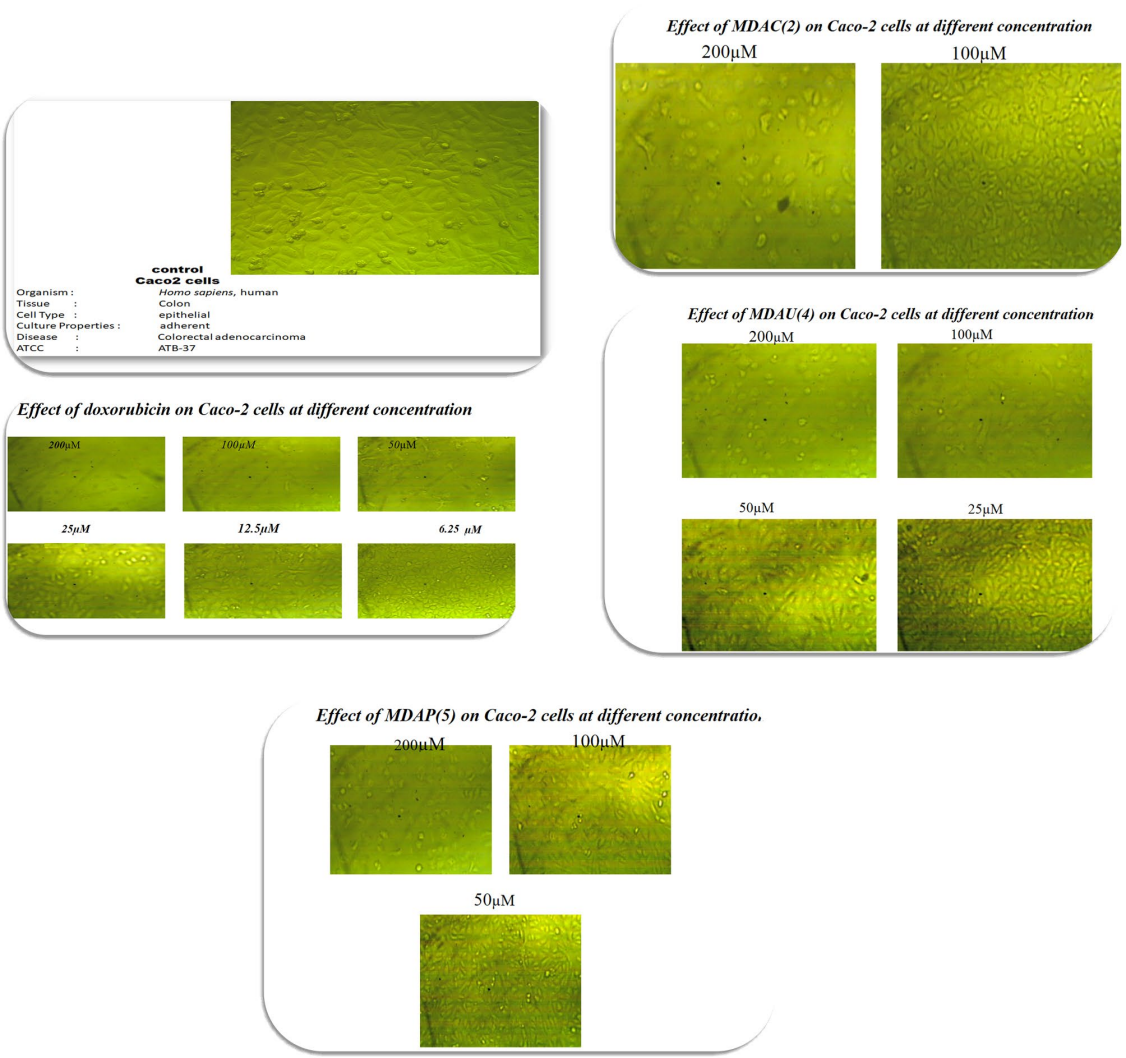
Effect of uracil pyrazole cellulose on **Caco2** cells with different concentrations.

Uracil pyrazole cellulose compound was investigated on the cell growth from two tumor cells (**A549** and **Caco2**) at (6.25, 12.5, 25, 50, 100, and 200 µM) concentrations via neutral red uptake analysis which is constructed on the ability of viable cells to include and bind the supravitally dye neutral red in the lysosomes. Doxorubicin (Dox) was used as a standard drug with IC_50_ values of 4.8 and 22.5 µM against tested cells **(A549** and **Caco2** respectively). The use of DMSO as a solvent had an insignificant effect on the viability of **A549** and **Caco2** cells when preserved for 48 h. Compared with control values, all heterocycles meaningfully affected cell growth inhibition. Data in Table 2 and Fig. 4 exposed that the cytotoxic activity of the heterocycles was in the descendant order of synthesized heterocycles **MDAU(4)** > **MDAC(2)** > **MDAP(5)** against **A549** cancer cells. At 48 h, compounds **MDAU(4)** (17.3 µM) showed more inhibitory effects compared with starting **MDAC(2)** (59 µM) while compound **MDAP(5)** (89.7 µM) exerted a slight cytotoxic influence on **A549** cells comparable with control values. Results explained in Fig. 5 presented a middling percentage of the toxicity of **Caco2** tumor cells preserved with different concentrations of cellulose compounds after 48 h. All data in Table 2 and Fig. 5 revealed that the cytotoxic activity from the highest to the lowest is as follows compound **MDAU(4)** > **MDAP(5)** > **MDAC(2)** contra **Caco2** cells. The treatment of **Caco2** cells with compounds **MDAU(4)** (89.26 µM) exhibited high cytotoxic activity as comparable with **MDAC(2)** (188.8 µM) after 48 h. Additionally, **MDAP(5)** with IC_50_ values of (150.24 µM) displayed moderate inhibition growth than **MDAC(2)** against **Caco2** tumor cells in comparison with control values. Lastly, these outcomes exhibited that the cellulosic compounds **MDAU(4)** revealed more inhibitory effect compared to starting material and standard values once preserved with two tumor cells (**A549** and **Caco2**) after 48 hours^43,44^.

**Table 2:** The cytotoxicity activity of heterocycles on **A549** and **Caco2** cells.

A549 cells		Caco2 cells	
Compounds	IC_50_ (µM)	Compounds	IC_50_ (µM)
MDAC(2)	59	MDAC(2)	188.8
MDAU(4)	17.3	MDAU(4)	89.26
MDAP(5)	89.7	MDAP(5)	150.24
Doxorubicin	4.8	Doxorubicin	22.54

IC_50_: Concentration required to inhibit cell viability through 50%.

### SAR investigation

**Figure 6:**
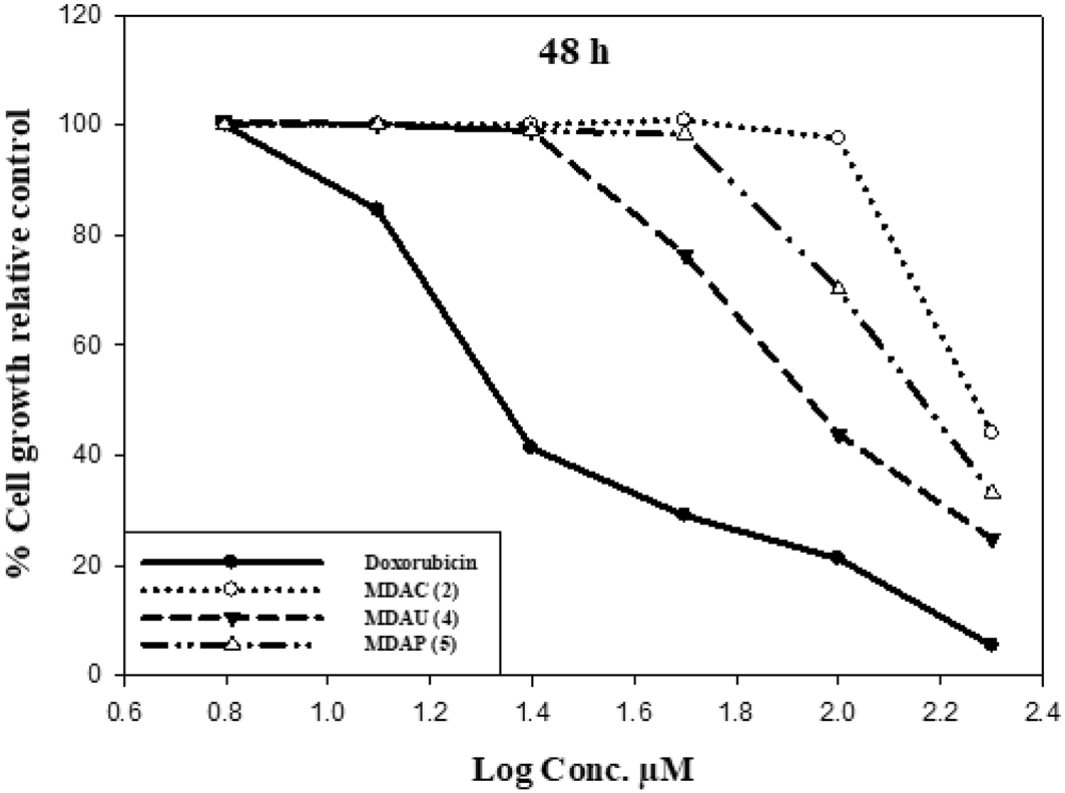
Effect of cellulosic compounds on **Caco2** cells at 48 h.

SAR examination showed the activity of the combination of heterocyclic moieties with cellulose compounds participated in variation in the cytotoxic effect of these compounds. At 48 h incubation time, The presence of dioxo tetrahydropyrimidinyl hydroxyl butenamide moiety in compound **MDAU(4)** is more effective and more cytotoxic than the presence of pyrimidinyl pyrazole carboxamide ring in **MDAP(5)** compared to the presence of dimethyl aminooxopentenenitrile ring in starting compound **MDAC(2)** when treated with **A549** and **Caco2** cells. These outcomes recognized the importance of the presence of uracil pyrazoles cellulosic compounds for anticancer activity as displayed in Fig. 6^45,46^.

### Antitumor docking analysis

**Figure 7:**
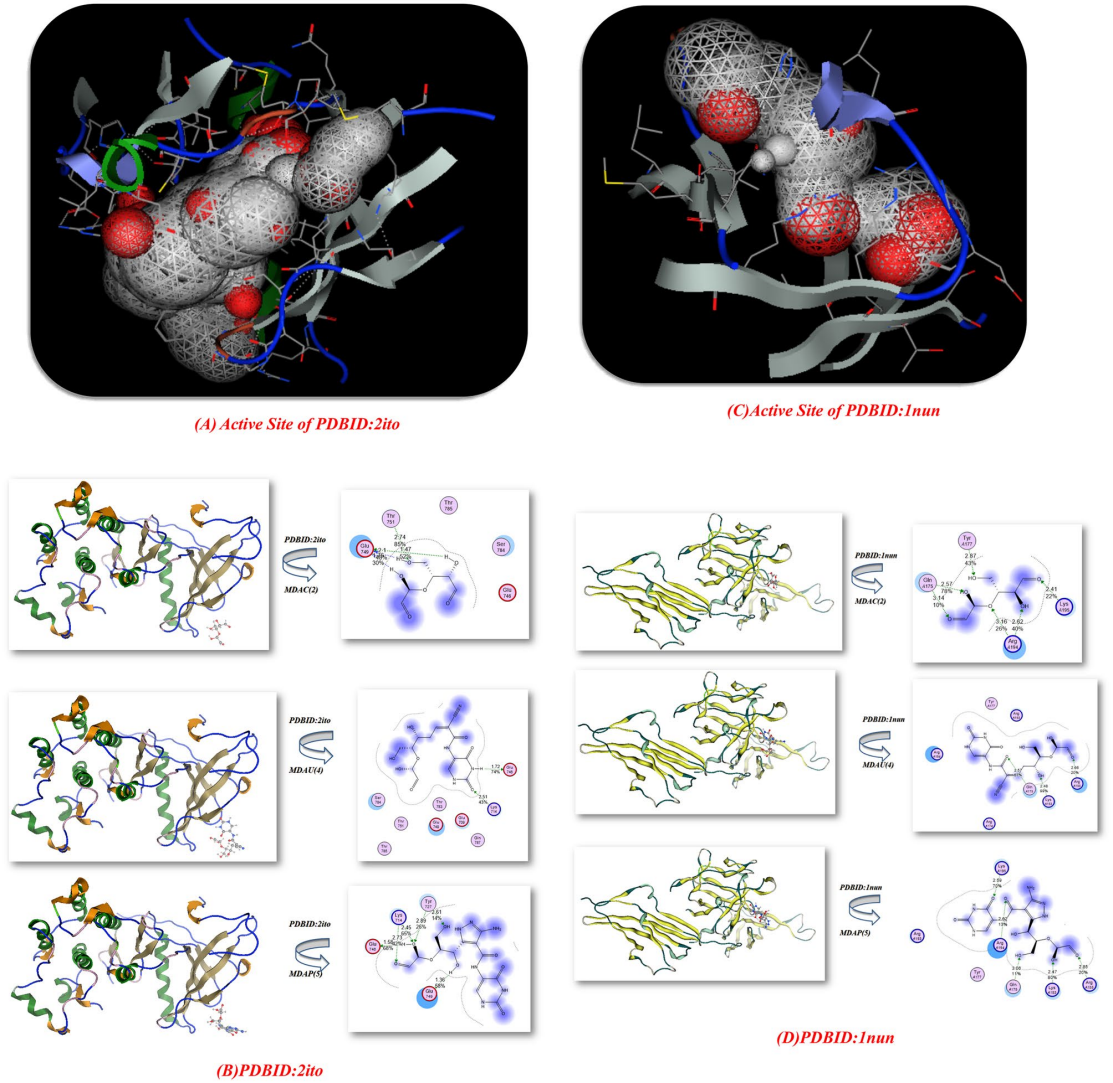
(**A**–**D**) Graphs of docking of active site and uracil pyrazole cellulosic with **PDBID:**2ito and **PDBID:**1nun; respectively.

Moreover, the docking simulation of uracil pyrazole cellulose was improved with bond lengths in Å units via the Moe program^30,47^. The less energies were then executed to preserve the geometrical optimization and systematic inquiries with an RMS gradient of 0.01 Å. Crystal structure of EGFR kinase domain G719S mutation in complex with Iressa (**PDBID:**2ito)^31^, crystal structure analysis of the FGF10-FGFR2b Complex (**PDBID:**1nun)^32^ as demonstrated which were occupied from protein data bank in Table 3 and Fig. 7**.** The active site of PDBID:2ito showed 95 sides and different residues of amino acids such as (Lys 716, Leu 718, Ser 719, Ser 720, Gly 721, Ala 722, Phe 723, Gly 724, Val 726, Lys 728, Ala 743, Ile 744) as showed in Fig. 7A**,** and the active site in protein **PDBID:1nun** showed 39 sides attached with amino acids such as (His 72, Leu 73, Gly 75, Asp 76, Arg 78, Arg 80, Ile 113, Thr 114, Ser 115, Glu 154, Ile 156, Gly 160, Asn 162, Leu 202, Pro 203) as displayed in Fig. 7C. In this docking simulation, some researchers have used in silico docking and catalytic site residue analysis to successfully identify and study the enzymes used in cellulose derivatives. In Fig. 7B the interaction between synthesized cellulosic derivatives with PDBID:2ito which showed excellent binding affinity MDAU(4) with − 10.325 kcal/mol and length distance (1.72, 2.51 Å) and attached amino acids (Glu 746, Lys 714, Gln 787, Glu 709, Thr 783, Glu 749, Thr 751), which is attached with N and C=O of uracil moiety, then the MDAP(5) showed − 10.052 kcal/mol and it showed the least distance 1.58, 2.61, 2.73, 2.89 Å (Tyr 727, Lys 714, Glu 746, Glu 749) due to presence of more NH groups which increase its interaction and electrostatic hydrogen bond interaction, while the MDAC(2) showed least binding affinity with − 9.84 kcal/mol and high length(2.1, 2.74 Å)Thr751, Glu 749, Thr 785, Ser 784, Glu 746 and this result due to absence of chelating site of NH or C=O of uracil which increase the binding efficiency site. Moreover, the docking simulation of cellulosic compounds with **PDBID:**1nun also showed the most reactivity of the binding site of MDAU(4) with − 9.612 kcal/mol, and bond lengths 2.51 Å and displayed different interaction with amino acids through electrostatic hydrogen bond interaction (Lys A153, Arg A135, Gln A175, Arg A174, Arg A1974). Furthermore, the MDAP(5) showed moderate activity in both proteins with energy − 8.524, − 10.052 kcal/mol; respectively for PDBID:2ito and 1nun and bond length 1.58–3.06 Å with amino acids (Thr751, Glu 749, Thr 785, Ser 784, Glu 746), (Tyr 727, Lys 714, Glu 746, Glu 749), (Tyr A177, Lys A195, Gln A175) and (Lys A1975, Lys A153, Arg A155, Gln A175, Tyr A177, Arg A193) which showed most interaction from OH of glycose of the cellulosic chain as presented in Fig. 7D.

**Table 3:** Docking analysis with PDBID**:2ito** and** 1nun.**

PDBID:2ITO					PDBID:1NUN		
	Affinity of Energy (kcal/mol)	Distance (Å)	Amino acids		Affinity of Energy (kcal/mol)	Distance (Å)	Amino acids
MDAC(2)	− 9.84	2.1, 2.74 Å	Thr 751, Glu 749, Thr 785, Ser784, Glu 746	MDAC(2)	− 8.9231	2.41,2.57,2.87 Å	Tyr A177, Lys A 195,Gln A175
MDAU(4)	− 10.325	1.72, 2.51 Å	Glu 746, Lys 714, Gln 787, Glu 709, Thr 783, Glu 749, Thr 751	MDAU(4)	-9.612	2.48, 2.66 Å	Lys A153, Arg A135, Gln A175, Arg A174, Arg A1974
MDAP(5)	− 10.052	1.58,2.61,2.73,2.89 Å	Tyr 727, Lys 714, Glu 746, Glu 749	MDAP(5)	− 8.524	2.47, 2.59, 2.85, 3.06 Å	Lys A1975, Lys A153, Arg A155, Gln A175, Tyr A177, Arg A193

From the above results, we concluded that the presence of uracil acetamide attached to cellulosic moiety increased the electrostatic hydrogen interaction and presence of NH, C=O enhanced this activity, and the presence of pyrazole uracil cellulosic derivatives enhanced the biological evaluation and docking result confirmed the biological evaluation.

## Molecular studies

**Figure 8:**
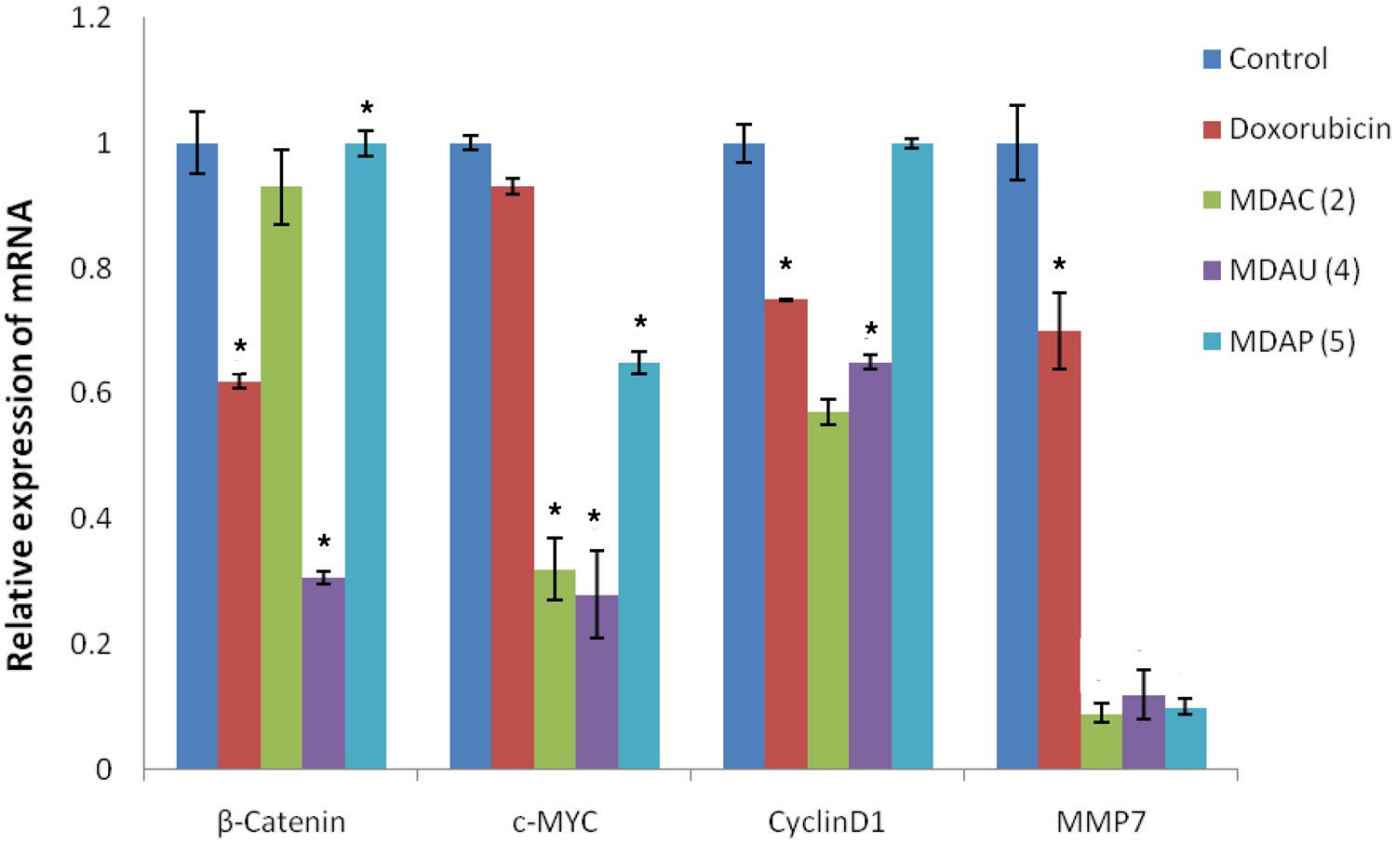
Effect of Doxorubicin, **MDAC(2), MDAU(4) and MDAP(5)** on levels of *β-Catenin, c-Myc, Cyclin D1 and MMP7* genes in **A549** cells. Data are represented as mean ± SEM, Data were reproducible, *P < 0.05.

The impact of **A549** cells preserved with uracil pyrazole cellulose **MDAC(2), MDAU(4), MDAP(5)** on mRNA expression levels *β-Catenin, c-Myc, Cyclin D1,* and *MMP7* genes were estimated utilizing IC_*50*_ values of these heterocycles after 48 hours and were estimated through calculating the percentage of its expression to that of *β-Actin* and in comparison to control values. From earlier analysis, it is fit recognized that the expression levels of *β-Catenin, Myc, Cyclin D1, and MMP7* are up-regulated in **A549** cells^48,49^. The Wnt signaling pathway is a complex pathway that regulates cell growth and proliferation^50^. The abnormal excitation of the pathway due to genetic mutation or increased stability can activate the abnormal expression of downstream target genes, including, c-Myc, Cyclin, and MMP-7, which can lead to cell proliferation, inhibition of cell apoptosis, and tumor formation^51^. Canonical Wnt/β-catenin pathway stimulates gene transcription through β-catenin^48,52^. MYC proto-oncogene amplification is closely related to tumor formation, development, and metastasis and is highly expressed in cervical cancer, breast cancer, gastric cancer, and other tumors^41^. Cyclin D1 considerably contributed to the development of the G1 to S phase^10,53^. The previous studies showed that the MMP-7 expression has been closely associated with tumor invasion and metastasis^5,10,41,54^. The current results showed that doxorubicin decreased significantly the expression levels of *β-Catenin, Cyclin D1, and MMP7* genes in **A549** cells as compared to control values (Fig. 8). On the other hand, treatment of **A549** cells with compounds **MDAC(2)** and **MDAP(5)** resulted in a significant reduction in levels of MMP7 and c-MYC genes in comparison with control values. Besides, compound **MDAU(4) **exhibited a significant reduction in levels of β-Catenin, c-Myc, Cyclin D1, and MMP7 genes in A549 cells compared to control values (Fig. 8). From obtained results, we found that compound **MDAU(4)** is the most promising anticancer agent against A549 cancer cells through the reduction of expression levels of β-Catenin, c-Myc, Cyclin D1, and MMP7 genes compared to control values^41,50^.

### Genetics docking calculation

**Figure 9:**
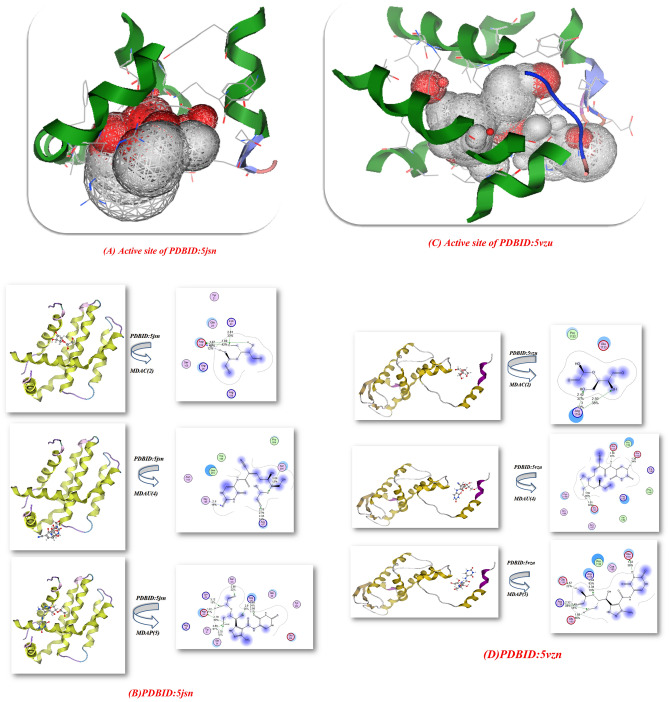
(**A**–**D**): Graphs of docking stimulation of MDAC(2), MDAU(4) and MDAP(5) with different proteins.

Additionally, uracil pyrazole cellulosic were interact with two types of proteins, particularly for genes for instance Bcl2-inhibitor complex **PDBID:** (5jsn)^33^ and Crystal structure of the Skp1-FBXO31-cyclin D1 complex **PDBID:** (5vzu)^34^; respectively. Figure 9A–D and Table.4,

**Table 4:** Docking uracil cellulose heterocycles with **PDBID:5jsn, 5vzu.**

PDBID:5jsn					**PDBID:5vzu**		
	Affinity of Energy (kcal/mol)	Distance(Å)	Amino acids		Affinity of Energy (kcal/mol)	Distance(Å)	Amino acids
***MDAC(2)***	-8.32	1.67, 1.69, 2.81 Å	Lys 22, Gln 25, Asp 102, Arg 26,	***MDAC(2)***	-9.1023	2.42, 2.59 Å	Glu 1133, Arg 1154, Pro 1132
***MDAU(4)***	-9.412	2.9, 2.76 Å	Ser205, Arg 107, Gly 145, Leu 201, Trp 144, Pro 204	***MDAU(4)***	-9.5651	1.59, 1.38 Å	Glu 1161, Glu 1150, Glu 1133, Arg 1154, Arg 1136, Phe 1145
***MDAP(5)***	-8.724	1.91, 2.75, 2.68, 2.9 Å	Arg 106, Ser 105, Lys 22, Asp 102, Tyr 21, Gln 25, Gln 99, Tyr 202, Glu 209	***MDAP(5)***	-10.5147	1.42, 2.44, 1.68, 2.32 Å	Glu 1133, Arg 1154, Arg 1136, Glu 1150, Glu 1161, Cys 1160, Pro 1132

The docking analysis of PDBID:5jsn showed 50 sides with different residues (Tyr 18, TYP 21, LYS22, Gln 25, Arg 26, GLY101, Asp102, SER 105, ARG 106, ARG 109, PHE 112, ALA113, SEP 116, GLU52) which is uracil pyrazole cellulosic displayed excellent outcome with MDAU(4) with high binding with − 9.412 eV and shortage bond length (2.9, 2.76 Å) and dissimilar amino acids (Ser205, Arg 107, Gly 145, Leu 201, Trp 144, Pro 204) and the MDAP(5) showed − 8.724 kcal/mol and its length(1.91, 2.75, 2.68, 2.9 Å) and linked with (Arg 106, Ser 105, Lys 22, Asp 102, Tyr 21, Gln 25, Gln 99, Tyr 202, Glu 209) which attached with OH and C=O and MDAC(2) showed least binding affinity with − 8.32 kcal/mol with 1.67, 1.69, 2.81 Å and amino acids Lys 22, Gln 25, Asp 102, Arg 26 which indicate the presence of these cellulosic heterocyclic inside the pocket of protein. Moreover, the interaction of cellulosic heterocyclic with PDBID:5vzu showed 75 sides of reactivity with amino acids (Leu 29, Leu 32, THR 39, LYS 42, PHE 43, ASP44, ARG 45, HIS 64, THR 67) (Fig. 9c) which most action with MDAP(5) with − 10.5147 kcal/mol and shortage length (1.42, 2.44, 1.68, 2.32 Å) and different acids Glu 1133, Arg 1154, Arg 1136, Glu 1150, Glu 1161, Cys 1160, Pro 1132 and attached with regarded for OH group of glycoside ring and C=O, NH of uracil and NH_2_ of pyrazole ring as demonstrated in Fig. 9D, while MDAU(4) showed moderate with − 9.5651 kcal/mol and its length(1.59, 1.38 Å)Glu 1161, Glu 1150, Glu 1133, Arg 1154, Arg 1136, Phe 1145, while the MDAC(2) showed least bonded to the pocket of protein this indication that the modification of glycose linkage increase the binding affinity inside pocket of proteins and increase its stability with different amino acids as demonstrated in Fig. 9D.

## Theoretical analysis

### Monomers of cellulosic heterocycles optimization

**Figure.10:**
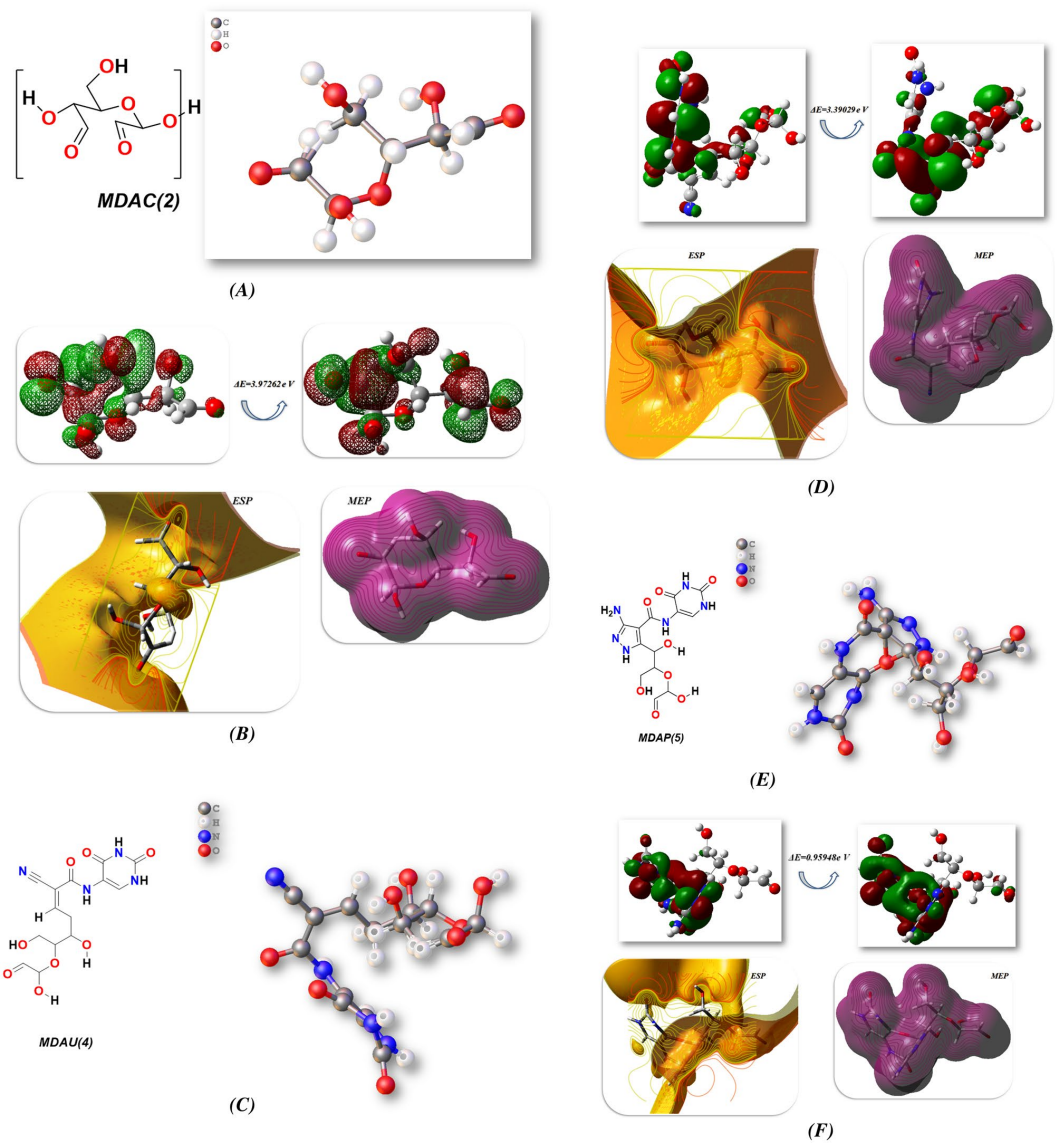
(**A**–**F**): FMO of an optimised chemical structure. DFT/B3LYP/6–311 (**G**) level was used by MDAC(2), MDAU(4), and MDAP(5) for ESP and MEP.

The Gaussian(09) program was used for uracil pyrazole heterocycle optimization^36,55,56^ through DFT/B3LYP/6-311(G) level. Besides, physical features of molecular structures of MDAC(**2**), MDAU(**4**), and MDAP(**5**) were regarding (σ) softness^57^, (χ) electronegativities^58^, (ΔN_max_) electronic charge^59^, (η) hardness, (ω)^60^ electrophilicity^61^, (S) softness^62^, and (Pi) chemical potential^63,64^, from the equations (1^_^8) which were listed in Table 5 and Fig. 10^59^

1 $$\Delta E = E_{LUMO} - E_{HOMO}$$

2 $$\chi = \frac{{ - (E_{HOMO} + E_{LUMO} }}{2}$$

3 $$\eta = \frac{{(E_{HOMO} - E_{LUMO} )}}{2}$$

4 $$\sigma = {1}/\eta$$

5 $${\text{Pi}} = - \chi$$

6 $${\text{S }} = {1}/{2}\eta$$

7 $$\omega = {\text{ Pi}}^{{2}} /{2}$$

8 $$\Delta {\text{N max}} = - {\text{ Pi}}/\eta$$

**Table 5:** Computed physical parameters for synthesized cellulosic heterocycles.

Physical parameters	MDAC(2)	MDAU(4)	MDAP(5)
E_T_ (au)	− 685.498	− 1363.21738	− 1434.5892
E_HOMO_(eV)	− 5.81811	− 6.145739	− 3.06756
E_LUMO_ (eV)	− 1.84549	− 2.75544	− 2.10808
ΔE (ev)	3.97262	3.39029	0.95948
µ (Debye)	2.9272	12.1785	2.5425
χ (eV)	3.832	4.451	2.588
η (eV)	1.986	1.695	0.480
σ (eV)	0.503	0.590	2.084
Pi (eV)	− 3.832	− 4.451	− 2.588
S (eV)	0.252	0.295	1.042
ω (eV)	3.696	5.842	6.980
ΔN max	1.9295065	2.62595	5.3916666

Moreover, the uracil heterocycles exhibited the non-planarity with DFT/B3LYP/6-311(G) level, and MDAC(2) showed total energy of it was (− 18,653.3596 eV)(− 430,156.581 kcal/mol) with coplanarity of two carbonyl group in glycoside ring and they were far of each other and blinded with each other with hydrogen bonds which make the band energy gap with ∆E = 3.97262 eV and this high gap gave the aldehyde stability and ability to interact again with other compound and as demonstrated in Fig. 10A,B the delocalization of electrons in HOMO and LUMO were in all MDAC(2) and indicate of reactivity of it. Furthermore, the dipole moment of MDAC(2) showed that 2.9272D can simply for energy departure and provided it capability to react once more, its electronegativity which designated the affinity of an atom to cooperate with mutual pair of electrons exhibited a high value of 3.832 eV and its hardness and softness showed low value with 1.986 eV and 0.503; respectively and it due to ability to change its electron cloud surrounding the HC=O of aldehyde group. The Pi chemical potential of MDAC(2) gives it the capability to respond and captivate more energy in dissimilar temperature varieties and they presented − 3.832 eV which gave them the capability to accumulate energy inside it. ω indicated electrophilic attractiveness and electron flow between donor and acceptor so, the MDAC(2) exhibited higher electrophilic attractiveness to captivate electrons with 3.696 eV^65,66^,

Additionally, the energy of the cellulosic compound MDAU(4) is more stable than MDAC(2) with (− 37,095.0520509 eV)( − 855,432.003784 kcal/mol) due to the presence of the uracil moiety connected to MDAC(2) and more NH groups, which increase the stability of the molecule^35,42^. This is demonstrated in Fig. 9C,D. Additionally, FMO represents electron affinity and ionization potential. Additionally, the heterocycle's stability and reactivity were determined by the band gap energy. While heterocycles with a smaller band gap will be less stable and more reactive, those with a larger band gap will be more stable^67–69^, According to Fig. 9B, the HOMO–LUMO orbitals' energy planes and circulations were computed at the B3LYP/6-311G(d, p) level for the complex MDAU(4) on the glucose and uracil moiety in HOMO and LUMO due to more NH and OH groups, which provided it stability with a gap energy of 3.39029 eV. With a dipole moment of 12.1785D, this compound has a high dipole attraction that makes charge separation simple. Due to the presence of CN–CH=, which gives an atom the ability to respond again, practical MDAU(4) displayed a high value of 4.451 eV for its electronegativity, and similarly, the (eV) can easily change the electron charge with − 4.451 eV can be easily to store more energy inside it^70–73^. Due to the formation of pyrazole attached to cellulose with (− 39,037.179122 eV)( − 900,218.50658 kcal/mol), its HOMO–LUMO delocalization of charge on pyrazole and uracil rings and lack of delocalization on OH group of glycose ring, as well as the presence of NH and carbonyl of pyrazole uracil, the MDAP(5) demonstrated greater stability than MDAU(4), gave it stability, its band energy gap was 0.95948 eV, and the charge energy departure of its dipole moment was 2.5425D. As a result, the MDAP(5) displayed high electrophilic attractiveness with 6.980 eV and electronic charge ΔN max with 5.39 eV due to more NH and NH_2_, which increased the electronic charge^74,75^ on it as shown in Table.5 and Fig. 10(E, F).

## Conclusion

In this elucidation, the cellulosic aldehyde was combined with uracil acetamide to produce the corresponding arylidene cellulosic derivatives, which served as the active site for the nucleophilic addition to give the corresponding pyrazole cellulosic derivative. These heterocycles were then confirmed through various analyses, and it was discovered that more hydrogen bond interactions caused the binding to occur on the cellulosic surface. Additionally, the tested compound MDAU(4) displayed the lowest IC_50_ values and the largest cytotoxic activity against A549 and Caco2 cells in comparison to the beginning compound MDAC(2)^47^. And also, it was a reduced expression levels of the Wnt genes (β-Catenin, c-Myc, Cyclin D1, and MMP7) are likewise expressed at lower levels in A549 cells after 48 h. Additionally, the docking stimulation revealed that MDAU(4) had an excellent binding affinity for the majority of proteins with the least amount of energy required due to the presence of more NH and OH in its structure. These findings were further supported by the physical descriptors of these cellulosic compounds, which demonstrated the stability of these compounds due to increased hydrogen bonding interaction.

## Data Availability

All data generated or analyzed during this study are included in this published.
