# Author Correction: Anti-proliferative activity, molecular genetics, docking analysis, and computational calculations of uracil cellulosic aldehyde derivatives

**DOI:** 10.1038/s41598-024-54632-6

**Published:** 2024-02-28

**Authors:** Asmaa M. Fahim, Sawsan Dacrory, Ghada H. Elsayed

**Affiliations:** 1 Green Chemistry Department, National Research Centre (NRC), P.O. Box 12622, Dokki, Cairo, Egypt; 2 Cellulose and Paper Department, National Research Centre, P.O. Box 12622, Giza, Egypt; 3 Department of Hormones, National Research Centre (NRC), P.O. Box 12622, Dokki, Giza, Egypt; 4 Stem Cells Lab, Center of Excellence for Advanced Sciences, National Research Centre (NRC), P.O. Box 1262, Dokki, Giza, Egypt

Correction to: *Scientific Reports* 10.1038/s41598-023-41528-0, published online 04 September 2023

The original version of this Article contained errors.

In the original version of this Article, the β-Catenin gene and the c-Myc gene were misspelt as ‘catenin gene’ and ‘Myc’.

As a result, in the Abstract section,

“After 48 h, Compound MDAU(4) had a stronger inhibitory effect on the growth of A549 and Caco2, compared to standard values. Then, using QRT-PCR, the appearance sites of the genes -Catenin, c-Myc, Cyclin D1, and MMP7 in A549 cells were examined. By reducing the expression levels of the Wnt signaling cascade genes -Catenin, c-Myc, Cyclin D1, and MMP7 when administered to A549 cells, compound MDAU(4) was shown in this investigation to be a viable candidate compared to lung cancer.”

now reads,

“After 48 h, Compound MDAU(4) had a stronger inhibitory effect on the growth of A549 and Caco2, compared to control cells. Then, using QRT-PCR, the appearance sites of the genes β-Catenin, c-Myc, Cyclin D1, and MMP7 in A549 cells were examined. By reducing the expression levels of the Wnt signalling cascade genes (β-Catenin, c-Myc, Cyclin D1, and MMP7) when administered to A549 cells, compound MDAU(4) was shown in this investigation to be a viable candidate compared to lung cancer.”

In the Introduction,

“Additionally, we examined these cellulosic derivatives with cytotoxic effects on lung cancer cells (A549) and colon cancer cells (Caco2), and we then examined the levels of -Catenin, Myc, Cyclin D1, and MMP7 gene expression in A549 cells.”

now reads,

“Additionally, we examined these cellulosic derivatives with cytotoxic effects on lung cancer cells (A549) and colon cancer cells (Caco2), and we then examined the levels of β-Catenin, c-Myc, Cyclin D1, and MMP7 gene expression in A549 cells.”

Under the Molecular studies section

“The **A549** cells preserved with uracil pyrazole cellulose **MDAC(2), MDAU(4), MDAP(5)** on mRNA expression levels *β-Catenin, Myc, Cyclin D1,* and *MMP7* genes were estimated utilizing IC*50* standards of these heterocycles later 2 days and were estimated through calculating the percentage of its expression to that of *β-Actin* and in comparison to control values. From earlier analysis, it is fit recognized that the expression levels of *β-Catenin, Myc, Cyclin D1, and MMP7* are up-regulated in **A549** cells^48,49^. The Wnt signaling pathway is a complex pathway that regulates cell growth and proliferation50. The abnormal excitation of the pathway due to genetic mutation or increased stability can activate the abnormal expression of downstream target genes, including, Myc, Cyclin, and MMP-7, which can lead to cell proliferation, inhibition of cell apoptosis, and tumor formation^51^.”

now reads,

“The impact of **A549** cells treated with uracil pyrazole cellulose **MDAC(2), MDAU(4), MDAP(5)** on mRNA expression levels *β-Catenin, c-Myc, Cyclin D1,* and *MMP7* genes were estimated utilizing IC_50_ values of these heterocycles later 2 days and were estimated through calculating the percentage of its expression to that of *β-Actin* and in comparison to control values. From earlier studies, it is fit recognized that the expression levels of *β-Catenin, c-Myc, Cyclin D1, and MMP7* are up-regulated in **A549** cells^48,49^. The Wnt signaling pathway is a complex pathway that regulates cell growth and proliferation_50_. The abnormal excitation of the pathway due to genetic mutation or increased stability can activate the abnormal expression of downstream target genes, including, c-Myc, Cyclin, and MMP-7, which can lead to cell proliferation, inhibition of cell apoptosis, and tumor formation^51^.”

and where

“Besides, compound **MDAU(4)** exhibited a significant reduction in levels of β-Catenin, Myc, Cyclin D1, and MMP7 genes in A549 cells compared to control values (Fig. 8). From obtained results, we found that compound **MDAU(4)** is the most promising anticancer agent against A549 cancer cells through the reduction of expression levels of β-Catenin, Myc, Cyclin D1, and MMP7 genes compared to control values^41,50^.”

**Figure 8:**
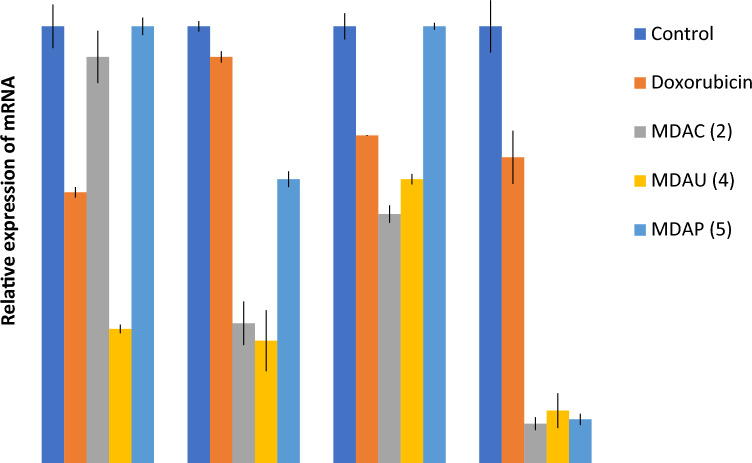
Effect of Doxorubicin, **MDAC(2), MDAU(4) and MDAP(5)** on levels of *β-Catenin, c-Myc, Cyclin D1 and MMP7* genes in **A549** cells. Data are represented as mean ± SEM, Data were reproducible, *P < 0.05.

now reads,

“Besides, compound **MDAU(4)** exhibited a significant reduction in levels of β-Catenin, c-Myc, Cyclin D1, and MMP7 genes in A549 cells compared to control values (Fig. 8). From obtained results, we found that compound **MDAU(4)** is the most promising anticancer agent against A549 cancer cells through the reduction of expression levels of β-Catenin, c-Myc, Cyclin D1, and MMP7 genes compared to control values^41,50^.”

Finally, in the Conclusion

“The Wnt genes (-Catenin, Myc, Cyclin D1, and MMP7) are likewise expressed at lower levels in A549 cells after 48 h.”

now reads,

“And also, it was a reduced expression levels of the Wnt genes (β-Catenin, c-Myc, Cyclin D1, and MMP7) in A549 cells after 48 h.”

In addition, in the original version of Fig. 8, the statistical analysis did not contain error bars of SEM and stars of significance, and the gene names were not mentioned on the X-axis. The incorrect Fig. 8 and its accompanying legend appear below.

The original version of this Article has been corrected.

